# DNA barcoding of mayflies (Insecta: Ephemeroptera) from South India

**DOI:** 10.1080/23802359.2016.1219623

**Published:** 2016-09-04

**Authors:** Chellappa Selvakumar, Kumbakonam Govindarajaiyer Sivaramakrishnan, Sundaram Janarthanan

**Affiliations:** a Department of Zoology, University of Madras, Chennai, Tamil Nadu, India;; b Department of Zoology, Madras Christian College, Chennai, Tamil Nadu, India

**Keywords:** Ephemeroptera, COI, DNA barcoding, identification, South India

## Abstract

In this study, DNA barcodes were generated for 40 species belonging to 32 genera under 10 families of Ephemeroptera from South India. Nucleotide sequence divergences were calculated using the Kimura two-parameter distance model and a neighbour-joining analysis was performed to provide a graphic display of the patterns of divergence among the species. This study demonstrates that COI barcoding is effective in discriminating among the mayfly species of South India, and provides a reference library for their future molecular identification.

Mayflies are an archaic lineage of insects, dating back to the late Carboniferous or early Permian periods, some 290 mya (Brittain & Sartori 2003). They occupy freshwater and brackish water habitats across the world, with the exception of Antarctica. They constitute an important part of the food chain, mainly consuming primary producers such as algae and plants, and as a food source for vertebrate predators like fish. They are excellent biological indicators of water quality and habitat quality (Sivaramakrishnan et al. 1996; Buffagni 1997; Selvakumar et al. 2014). They are ideal objects for integrated phylogenetic, biogeographic and phylogeographic studies, being endowed with several archaic traits in all life stages along with rather weak dispersal powers. Many of the montane mayflies, both nymphs and imagos are equally charismatic. Nymphs are important for freshwater ecological and biomonitoring studies, but difficulties in their species identification level impede research.

DNA barcoding can contribute to speeding up local biodiversity assessments to prioritise conservation areas or to evaluate the success of conservation actions and provide information about evolutionary histories (Krishnamurthy & Francis 2012). The application of DNA barcoding to freshwater biomonitoring has generated much interest for several reasons (Hajibabaei et al. 2011; Pilgrim et al. 2011; Sweeney et al. 2011). DNA barcodes have also implied in studying the systematics, diversity, ecology, biogeography, and conservation of aquatic insects (Sivaramakrishnan et al. 2014; Gattolliat et al. 2015). A comprehensive barcode library has been established for mayflies from Canada, Mexico, and the United States (Ball et al. 2005; Zhou et al. 2009, 2010; Webb et al. 2012; Gattolliat et al. 2015). To our knowledge, no molecular work of this kind was undertaken on mayflies in India so far. The emerging trends in molecular systematics and molecular phylogeny of mayflies are quite evident from the review by Sivaramakrishnan et al. (2011). Our general aim is to develop a strategy for rapid construction of regional barcode libraries, and specific aim is to examine the efficiency of DNA barcoding for differentiating morphospecies. Present study deals with nymphs of mayflies due to their importance in freshwater ecology and for their biomonitoring value.

Mayfly nymphs were collected from stream and river basins of South India. The collected specimens were identified using scattered Indian mayfly taxonomic literature, under a stereo-zoom microscope. Samples used in this study included 44 specimens representing 40 species belonging to 32 genera and 10 families of Ephemeroptera from South India. Thirty-eight species were represented by single specimens, and 2 species characterized by more than one specimens (Table 1). DNA was extracted using DNeasy Blood and Tissue kit (Qiagen, Hilden, Germany). The mtCOI gene was amplified using universal primer LC01490 and HC02198 (Folmer et al. 1994). Sequencing was performed commercially by Amnion Biosciences Pvt. Ltd (Bangalore, India). Forward and reverse sequencing reads were assembled and corrected using BioEdit (Carlsbad, CA) and aligned using CLUSTALW (Cambridgeshire, UK). Neighbour-joining (NJ) tree and intraspecific and interspecific genetic divergence values were performed based on the Kimura 2-parameter (K2P) model using MEGA 5 (Tamura et al. 2011).

**Table 1.** Details of sample used in this study.

Family	Genus and species	Locality	Latitude/longitude	GenBank Accession No.	Barcode ID
Baetidae	1. *Acentrella vera* Muller-Libenau, 1982	Ramanathi	08°84′80″ N/77°31′40″ E	LC056072	MCSIM023-15
	2*. Acentrella vera* Muller-Libenau, 1982	Kannupulimettu	08°56′20″ N/77°12′25″ E	LC056071	–
	3. *Baetis* sp.	Ramanathi	08°84′80″ N/77°31′40″ E	LC061859	MCSIM025-15
	4. *Baetis michaelohubbardi* (Selva-Kumar, Sundar and Sivaramakrishanan, 2012)	Bhavani river	11°03′56″ N/76°32′14″ E	LC061856	MCSIM024-15
	5. *Chopralla ceylonensis* (Muller-Liebenau, 1983)	Pilavakal Dam	09°63′18″ N/77°51′93″ E	LC061854	MCSIM026-15
	6. *Cloeodes soldani* (Muller-Liebenau, 1983)	Ramanathi	08°84′80″ N/77°31′40″ E	LC061855	MCSIM027-15
	7. *Cloeon bicolor* Kimmins, 1947	Alwarkurichi	08°47′05″ N/77°24′07″ E	LC061857	MCSIM032-15
	8. *Labiobaetis jacobusi* Kubendran and Balasubramanian, 2015	Moolaiyar	10°05′01″ N/77°14′55″ E	LC056075	MCSIM028-15
	9. *Labiobaetis soldani* Kubendran, Balasubramanian and Sivaramakrishanan, 2014	Sivasailam	08°78′84″ N/77°34′72″ E	LC056076	MCSIM029-15
	10. *Nigrobaetis paramakalyani* Kubenderan and Balasubramanian, 2015	Ramanathi	08°84′80″ N/77°31′40″ E	LC056073	MCSIM030-15
	11. *Procloeon* sp.	Shenpagathoppu	09°36′36″ N/77°32′14″ E	LC061858	MCSIM033-15
	12. *Tenuibaetis frequentus* (Muller-Liebenau and Hubbard, 1985)	Kurangani	10°05′01″ N/77°14′55″ E	LC056074	MCSIM031-15
Caenidae	13. *Caenis* sp.	Alwarkurichi	08°47′05″ N/77°24′07″ E	LC061847	MCSIM034-15
	14. *Clypeocaenis bisetosa* Soldan, 1978	Alwarkurichi	08°47′05″ N/77°24′07″ E	LC061848	MCSIM035-15
Ephemerellidae	15. *Torleya nepalica* Allen and Edmunds, 1963	Papanasam	08°42′37″ N/77°22′03″ E	LC061850	MCSIM036-15
Ephemeridae	16. *Ephemera* (*Aethephemera*) *nadinae*	Jogigundi falls	13°29′55″ N/75°06′10″ E	LC061852	MCSIM037-15
Heptageniidae	17. *Afronurus kumbakkaraiensis* Venkataraman and Sivaramakrishnan, 1989	Adavinayinar	09°07′96″ N/77°23′19″ E	LC061844	MCSIM014-15
	18. *Epeorus petersi* Sivaruban, Venkataraman and Sivaramakrishnan, 2013	Kannupulimettu	08°56′20″ N/77°12′25″ E	LC061845	MCSIM015-15
	19. *Thalerosphyrus flowersi* Venkataraman and Sivaramakrishnan, 1987	Ramanathi	08°84′80″ N/77°31′40″ E	LC061846	MCSIM016-15
Leptophlebiidae	20. *Choroterpes* (*Choroterpes*) *petersi* Tong and Dudgeon, 2003	Bhavani river	11°03′56″ N/76°32′14″ E	LC061861	MCSIM005-13
	21. *Choroterpes* (*Euthraulus*) *alagarensis* Dinakaran, Balachandran and Anbalagan, 2009	Pilavakal Dam	09°63′18″ N/77°51′93″ E	LC061463	MCSIM006-13
	22. *Choroterpes*(*Euthraulus*) *nambiyarensis* Selvakumar, Arunachalam and Sivaramakrishanan, 2012	Kalikesam River	08°25′03″ N/77°23′48″ E	LC061464	MCSIM007-13
	23. *Choroterpes* (*Monochoroterpes*) *nandini* Selvakumar and Sivaramakrishanan, 2015	Nandinihole	13°23′23″ N/77°10′47″ E	LC061465	MCSIM011-15
	24. *Edmundsula lotica* Sivaramakrishnan, 1985	Nandinihole	13°23′23″ N/77°10′47″ E	LC061466	MCSIM009-13
	25. *Indialis badia* Peters and Edmunds, 1970	Kodaikanal	10°16′15″ N/77°33′15″ E	LC061467	MCSIM003-13
	26. *Isca* (*Isca*) *purpurea* Gillies, 1951	Tada falls	13°60′25″ N/79°84′52″ E	LC061468	MCSIM010-13
	27. *Nathanella indica* Demoulin, 1955	Kodaikanal	10°16′15″ N/77°33′15″ E	LC061469	MCSIM012-15
	28. *Nathanella saraswathiae* Sivaramakrishnan, Venkataraman and Balasubramanian, 1996	Nambikovil	08°26′01″ N/77°29′55″ E	LC061470	MCSIM012-15
	29. *Notophlebia ganeshi* Kluge, 2014	Kunthipula river	11°27′43″ N/76°45′63″ E	LC061471	MCSIM013-15
	30. *Notophlebia jobi* Sivaramakrishnan and Peters, 1984	Srimane falls	13°23′14″ N/75°10′46″ E	LC061472	MCSIM002-13
	31. *Petersula courtallensis* Sivaramakrishnan, 1984	Nambikovil	08°26′01″ N/77°29′55″ E	LC061474	–
	32. *Petersula courtallensis* Sivaramakrishnan, 1984	Gadananathi	08°48′04″ N/77°18′05″ E	LC061475	MCSIM001-13
	33. *Petersula courtallensis* Sivaramakrishnan, 1984	Kannupulimettu	08°56′20″ N/77°12′25″ E	LC061476	–
	34. *Petersula courtallensis* Sivaramakrishnan, 1984	Kodaikanal	10°16′15″ N/77°33′15″ E	LC061477	–
	35. *Thraulus gopalani* Grant and Sivaramakrishnan, 1985	Kottumthalam	08°42′02″ N/77°21′34″ E	LC061473	MCSIM004-13
Neoephemeridae	36. *Potamanthellus caenoides* (Ulmer, 1939)	Silent Valley	11°06′49″ N/76°25′52″ E	LC061849	MCSIM038-15
Polymitarcyidae	37. *Languidipes corporaali* (Lestage, 1922)	S. T. Mankad	08°29′29″ N/77°17′35″ E	LC061851	MCSIM039-15
Teloganodidae	38. *Derlethina tamiraparaniae* Selvakumar, Jacobus and Sivaramakrishnan, 2014	Nambikovil	08°26′01″ N/77°29′55″ E	LC057263	MCSIM018-15
	39. *Dudgeodes palnius* Selvakumar, Jacobus and Sivaramakrishnan, 2014	Kannupulimettu	08°56′20″ N/77°12′25″ E	LC057264	MCSIM019-15
	40. *Indoganodes jobini* Selvakumar, Jacobus and Sivaramakrishnan, 2014	Silent Valley	11°06′49″ N/76°25′52″ E	LC057262	MCSIM017-15
	41. *Teloganodes kodai* Sartori, 2008	Gadananathi	08°48′04″ N/77°18′05″ E	LC057265	MCSIM020-15
	42. *Teloganodes sartorii* Selvakumar, Jacobus and Sivaramakrishnan, 2014	Killiyur falls	11°47′40″ N/78°11′59″ E	LC061860	MCSIM021-15
	43. *Teloganella indica* Selvakumar, Jacobus and Sivaramakrishnan, 2014	Nandinihole	13°23′23″ N/77°10′47″ E	LC057266	MCSIM022-15
Tricorythidae	44. *Sparsorythus gracilis* Sroka and Solan, 2008	Papanasam	08°42′37″ N/77°22′03″ E	LC061853	MCSIM040-15

The present study established DNA barcode for 40 species of mayflies from South India through Genbank and BOLD systems. This is the first report of DNA barcode to the 40 species of mayflies from South India. Species details and sequence and barcode information are available at BOLD Systems (www.barcodinglife.org, ‘Molecular characterization of South Indian mayflies’ project). The details of the species along with their GenBank Accession numbers and Barcode ID are given in Table 1. Mean interspecific divergences computed for 40 species of South Indian mayflies ranged from 0.143% to 0.466%. The mean of all interspecific divergences was computed as 0.301%. The low levels of interspecific divergence occurred between two species within a genus and between the genus, such as *Nathanella indica* and *Nathanella saraswathiae* (0.17%), *Cloedes soldani* and *Labiobaetis soldani* (0.18%), *Labiobaetis jacobusi* and *Cloeon bicolor* (0.19%), and *Choroterpes nambiyarensis* and *Indialis badia* (0.14%).

Relatively, intraspecific genetic divergences were observed in the branches corresponding to species complexes in the NJ tree, as in *Petersula courtallensis*, which were divided into two subclades. The genetic divergence ranged from 0.003% to 0.051%, suggesting that more than one species will be represented. There was no difference between the closely related species *Labiobaetis jacobusi* and *L*. *soldani*. Three species, namely *Baetis* sp., *Caenis* sp., and *Procloeon* sp. were morphologically very distinct and also the present barcoding study clearly distinguished from their closely related species. The results of the overall NJ analysis of distances among the samples of 40 species are summarized in Figure 1. The obtained results indicate that the portion of COI used as a DNA barcode effectively discriminates among mayfly species. It should be noted that the tree presented here is intended as a representation of the distance matrix only, and should not be interpreted as a phylogenetic hypothesis.

**Figure 1.**
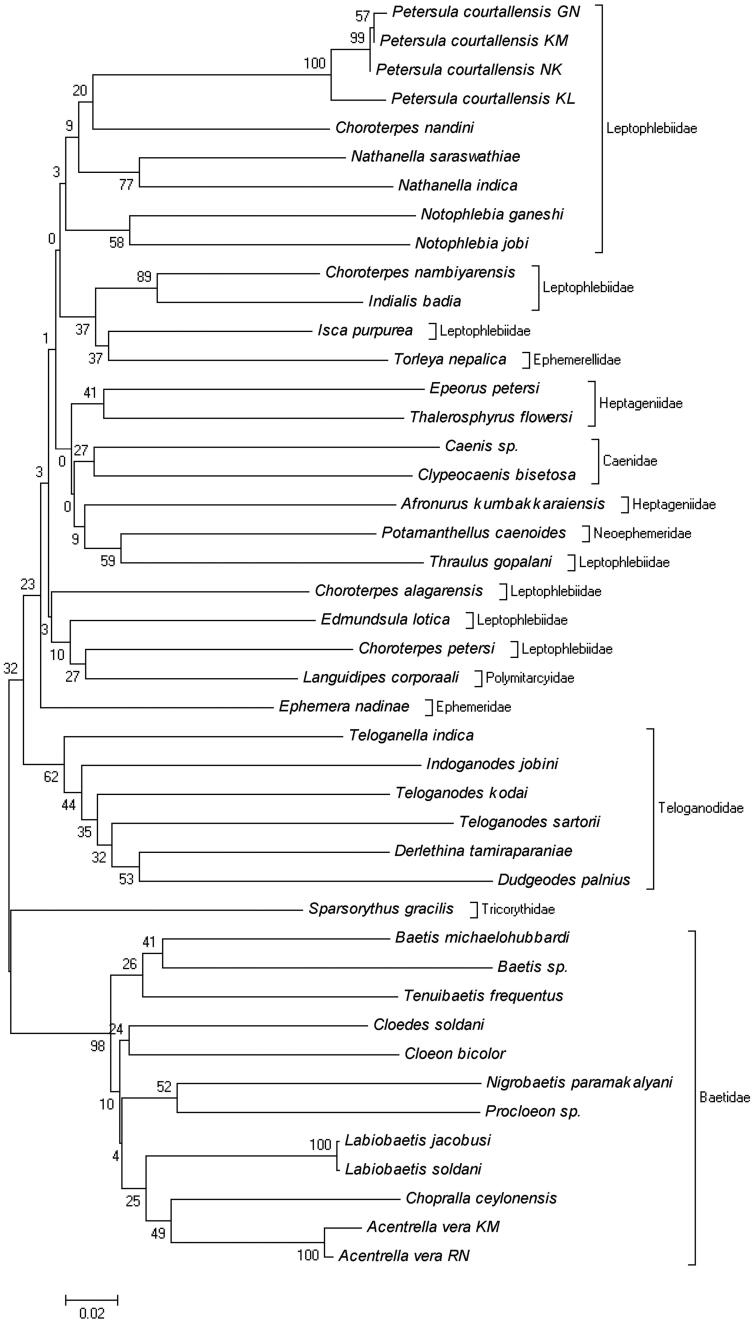
A Kimura 2-parameter NJ tree showing the DNA barcoding profile for 44 specimens of 40 nominal mayfly species from South India.

The present study reports for the first time COI barcode sequences for the purpose of species identification and the basis of global biodiversity assessment. All the species gave distinct COI sequences except *Labiobaetis jacobusi* and *L*. *soldani*, distinguishing them from conspecifics through the DNA barcode method (Figure 1). Detailed molecular analysis is required to differentiate *Labiobaetis jacobusi* and *L*. *soldani* using more samples. Minimum level of intraspecific genetic divergence were found in *Acentrella vera* (0.022%), though it is distributed over a very wide area within the Oriental Realm (Kluge et al. 2014). Maximum level of intraspecific genetic divergence was found in *Petersula courtallensis* ranging from 0.003% to 0.051%. In order to confirm this and to describe a new species, it will be necessary to perform detailed morphological and molecular studies. The possibility of the presence of cryptic species complex within the genus *Petersula* may not be ruled out. However, further detailed investigations are necessary to understand clearly the taxonomic situation of *Labiobaetis* species and *Petersula courtallensis*. *Baetis* sp., *Caenis* sp., and *Procloeon* sp. were very distinct species based on this barcoding study. It will be required to perform thorough morphological studies to describe the valid species. The NJ tree supported the results of previous studies that have found the COI barcode to be an effective tool for the identification in mayflies (Ball et al. 2005; Zhou et al. 2010).

The present study confirms that, DNA barcode can be used effectively for species identification of South Indian mayfly species although the success rates vary with the level of genetic structure and demographic history. DNA barcoding analysis represents an interesting approach to new studies of taxonomy and species recognition of South Indian mayflies as new species, including cryptic species. Also, DNA barcoding can be used to analyze a mayfly community to estimate species richness of an entire mayfly community and for further phylogeographic studies. The results indicate that more taxonomic and molecular work are required on Indian Ephemeroptera as many currently recognized species include several highly divergent, often polyphyletic, haplotypes, usually correlated with morphological differentiation among lineages.
